# PD-L1 imaging with [^99m^Tc]NM-01 SPECT/CT is associated with metabolic response to pembrolizumab with/without chemotherapy in advanced lung cancer

**DOI:** 10.1038/s41416-025-02991-w

**Published:** 2025-04-05

**Authors:** Daniel Johnathan Hughes, Gitasha Chand, Jessica Johnson, Ronan Tegala, Damion Bailey, Kathryn Adamson, Scott Edmonds, Levente K. Meszaros, Amelia Elizabeth Broomfield Moore, Thubeena Manickavasagar, Susan Ndagire, Spyridon Gennatas, Alexandros Georgiou, Sharmistha Ghosh, Debra Josephs, Eleni Karapanagiotou, Emma McLean, Hong Hoi Ting, James Spicer, Vicky Goh, Gary J. R. Cook

**Affiliations:** 1 Department of Cancer Imaging, School of Biomedical Engineering and Imaging Sciences, King’s College London, 5th Floor Becket House, London, UK; 2 King’s College London & Guy’s and St. Thomas’ PET Centre, St Thomas’ Hospital, London, UK; 3 Cancer Centre at Guy’s, Guy’s and St Thomas’ NHS Foundation Trust, London, UK; 4 Nanomab Technology (UK) Limited, Borehamwood, Hertfordshire UK; 5 Department of Nuclear Medicine, Guy’s and St Thomas’ NHS Foundation Trust, Guy’s Hospital, London, UK; 6 Department of Radiology, Guy’s and St. Thomas’ NHS Foundation Trust, St Thomas’ Hospital, London, UK; 7 King’s Health Partners Cancer Biobank, Cancer Centre at Guy’s, Guy’s and St Thomas’ NHS Foundation Trust, Great Maze Pond, London, UK; 8 School of Cancer and Pharmaceutical Sciences, King’s College London, Guy’s Campus, Great Maze Pond, London, UK; 9 Department of Histopathology, Guy’s and St Thomas’ NHS Foundation Trust, London, UK

**Keywords:** Molecular imaging, Diagnostic markers, Predictive markers, Cancer imaging

## Abstract

**Background:**

Programmed death-ligand 1 (PD-L1) immunohistochemistry is a predictive biomarker for anti-PD-(L)1 therapy in non-small cell lung cancer (NSCLC). It is not a reliable predictor of clinical benefit with non-invasive imaging providing a potential solution. We present the PECan study, the aim of which to assess the relationship of [^99m^Tc]-labeled anti-PD-L1 single-domain antibody (NM-01) single-photon emission computed tomography (SPECT)/CT with metabolic response to anti-PD-(L)1.

**Methods:**

PD-L1 tumour proportion score (TPS) measured using SP263 assay. [^99m^Tc]NM-01 SPECT/CT and [^18^F]FDG PET/CT performed before and 9-weeks following pembrolizumab with/without chemotherapy in patients with advanced NSCLC. Tumor (T) to blood pool (BP) maximum region of interest (ROI_max_) measurements performed in primary and metastatic lesions using SPECT/CT images.

**Results:**

Fifteen patients were included (median age 63 years, 9 male). Intertumoural heterogeneity evident in 10(67%) patients. Mean [^99m^Tc]NM-01 T:BP demonstrated moderate correlation with PD-L1 TPS (*r* = 0.45, *p* < 0.05). Depth of [^18^F]FDG PET/CT metabolic response at 9-weeks (*n* = 13), correlated strongly with baseline [^99m^Tc]NM-01 T:BP (*r* = −0.73, *p* < 0.05), but only moderately with PD-L1 TPS (*r* = −0.46, *p* = 0.06).

**Conclusion:**

[^99m^Tc]NM-01 SPECT/CT allows non-invasive quantification of PD-L1 in primary tumour and metastases in NSCLC. [^99m^Tc]NM-01 uptake moderately correlates with PD-L1 immunohistochemistry, determines heterogeneity, and is associated with early metabolic response to anti-PD-1 pembrolizumab.

**Clinical trials registration:**

PD-L1 Expression in Cancer (PECan) study (NCT04436406), registered 18 June 2020 https://clinicaltrials.gov/ct2/show/NCT04436406

## Background

Immune checkpoint inhibitors have revolutionised the treatment paradigm in non-small cell lung cancer (NSCLC). The co-inhibitory molecule programmed death-ligand 1 (PD-L1), expressed by tumour cells, downregulates effector T cells on interaction with programmed cell death protein 1 (PD-1) on their surface. Antibodies to PD-1 or PD-L1 (anti-PD-(L)1) interrupt this pathway, enabling immune recognition and promotion of cytotoxic T cell function. Anti-PD-(L)1 therapy alone or in combination with chemotherapy now represents a cornerstone of treatment in advanced NSCLC with significant improvements in both progression free and overall survival [1–4]. Up to around 20% of patients may have durable responses of several years, but a significant proportion of patients do not respond or maintain response to treatment [5]. Recent advances have also led to a number of anti-PD-(L)1 therapies being approved in the neo-/adjuvant settings, with improved pathological complete response rates and recurrence free survivals reported across several clinical trials [6, 7]. PD-L1 immunohistochemistry correlates with treatment response, particularly in advanced disease where PD-L1 tumour proportion score (TPS) ≥ 50% is associated with higher response rates and improved survival with single agent anti-PD-1 pembrolizumab compared to chemotherapy [1]. Whilst negative PD-L1 TPS < 1% is associated with a lesser benefit for single agent anti-PD-(L)1 therapy, around 10% of patients with advanced NSCLC and PD-L1 TPS < 1% may still demonstrate an objective and durable response [2]. In those patients with PD-L1 TPS negative (<1%) or low (1–49%), combination of anti-PD-(L)1 with chemotherapy has demonstrated improved survival compared to chemotherapy alone [4]. It remains controversial whether PD-L1 immunohistochemistry is the optimum biomarker of anti-PD-(L)1 response given these limitations [8]. One likely explanation is related to the spatial and temporal heterogeneity of PD-L1 expression, which is well documented, and is affected by a range of anti-cancer therapies [9–12]. Multiple biopsies throughout a patient’s cancer journey are clinically impractical and as such, novel non-invasive molecular imaging techniques of PD-L1 present a potential solution.

NM-01 is a small 15 kDa anti-PD-L1 camelid single-domain antibody (sdAb), which when labeled with technetium-99m ([^99m^Tc]) can be imaged using single-photon emission computed tomography (SPECT). Importantly, whilst NM-01 binds specifically to human PD-L1, preclinical studies have demonstrated that it does not interfere with atezolizumab (anti-PD-L1) monoclonal antibody nor PD-1 binding [13]. Unlike radionuclide labeled drug antibodies, it therefore has the potential to determine PD-L1 expression longitudinally in the presence of therapeutic anti-PD-(L)1 monoclonal antibodies, which have long half-lives and pharmacodynamic studies have demonstrated target occupancy in the region of months [14, 15]. The first-in-human study of [^99m^Tc]NM-01 demonstrated safety and acceptable dosimetry in 16 patients with NSCLC [16]. SPECT/CT imaging at 2 h post injection of [^99m^Tc]NM-01 demonstrated optimum tumour-to-background, with tumour-to-blood pool ratio (T:BP) correlating with PD-L1 immunohistochemistry.

Here we report the results of the PD-L1 expression in cancer (PECan) study, which involved imaging PD-L1 using [^99m^Tc]NM-01 SPECT/CT at baseline and 9-weeks following pembrolizumab (anti-PD-1), with or without chemotherapy, in patients with advanced NSCLC. The aims of the study included determining the intertumoural heterogeneity of [^99m^Tc]NM-01 uptake, its correlation with PD-L1 expression determined by immunohistochemistry, and its association with early metabolic response to anti-PD-1 therapy determined by fluorine-18 fluorodeoxyglucose positron emission tomography/computed tomography ([^18^F]FDG PET/CT) imaging.

## Methods

This single-centre, single-arm, open-label prospective biomarker study was conducted at Guy’s and St Thomas’ NHS Foundation Trust, and King’s College London, UK between November 2019 and September 2023. Eligibility criteria for the study included patients ≥ 18 years in age with advanced NSCLC scheduled to receive anti-PD-1/PD-L1 therapy with/without chemotherapy, with diagnostic tissue taken within 3 months prior to consent and with adequate quantity/quality for PD-L1 assessment. Exclusion criteria included those with a prognosis < 3 months and/or Eastern Cooperative Oncology Group (ECOG) performance status ≥ 2, previous immune checkpoint inhibitor therapy, systemic anti-cancer therapy within 14 days, radiotherapy to target lesion(s) within 42 days or palliative radiotherapy expected in the next 12 weeks, and pregnant/lactating female. The study was approved by a UK Research Ethics Committee and the Health Research Authority (IRAS reference 256684), all participants provided written informed consent and the study was conducted in accordance with the Declaration of Helsinki. The trial was registered at www.clinicaltrials.gov (NCT04436406).

Patients underwent PD-L1 imaging with [^99m^Tc]NM-01 SPECT/CT at baseline, i.e., prior to, and 9-weeks following standard-of-care anti-PD-1 therapy alone or in combination with chemotherapy. Additionally, [^18^F]FDG PET/CT imaging was performed at baseline and 9-weeks to determine metabolic response to anti-PD-1 therapy. Standard-of-care CT imaging was performed at baseline for TNM staging, and at 9- and 18-weeks for response assessment.

### Histopathological assessment

Diagnostic tissue was obtained prior to study enrolment, this included primary tumour (*n* = 5), single nodal metastasis (*n* = 5), multiple nodal metastases (*n* = 4), or non-nodal metastasis (*n* = 1). Hematoxylin and eosin (H&E) stained slides were assessed to confirm the histopathological diagnosis by an experienced lung pathologist according to local protocols. Where multiple sampled nodes contained malignant cells, a combined cell block was used for further analysis. Immunohistochemistry was performed, including PD-L1 TPS, which was assessed as per local laboratory guidelines using the Ventana PD-L1 (SP263) assay (Roche, Ventana Medical Systems Inc., Arizona, USA). A minimum of 100 viable tumour cells were required for analysis. The TPS was calculated as the percentage of PD-L1 positive tumour cells relative to the number of viable tumour cells in the sample as per manufacturers guidelines [17].

### [^99m^Tc]NM-01 radiopharmaceutical preparation

[^99m^Tc]NM-01 was synthesised using current Good Manufacturing Practice (cGMP) ingredients in our accredited radiopharmacy at the Nuclear Medicine Department at Guy’s Hospital, London. ^99m^Tc-triaquatricarbonyltechnetium(I) [^99m^Tc(OH_2_)_3_(CO)_3_]^+^ intermediate (pH 7.0–8.0) was added to 200 μg NM-01 in 100 μL phosphate-buffered saline (pH 7.4). This mixture was incubated at 37 °C for 1 hr to give [^99m^Tc]NM-01, which was subsequently diluted in physiological saline to 2.0 mL and passed through two 0.22 mm filters, prior to quality control testing. Final product with radiochemical purity > 90%, pH 6-9, endotoxin levels ≤ 175 EU/mL, and a colorless clear appearance was accepted for patient administration within a 6-hour shelf-life.

### [^99m^Tc]NM-01 SPECT/CT (PD-L1) scan

Patients were administered a median activity of 596 MBq (range 329–721) [^99m^Tc]NM-01, corresponding to 40–100 μg NM-01, intravenously. Dosing was selected according to microdosing principles and as determined by pre-clinical and phase 1 studies [13, 16]. Whole-body planar imaging and thoracic SPECT/CT were performed 2 h post-injection; the optimal time for acquisition previously determined in the reported phase I trial [16]. [^99m^Tc]NM-01 SPECT/CT was performed prior to and 9-weeks following initiation of anti-PD-1 with or without chemotherapy. Single field of view thoracic SPECT/CT imaging was performed on a Siemens Symbia Intevo Bold SPECT/CT scanner with low energy high-resolution collimators 256 × 256 matrix, 128 projections (64 views) over 180° rotation, at 20 s per projection. A low dose CT scan (at 110 kV, 25 mA, CTDI average 5.55 mGy, DLP average 246 mGy.cm) was performed for anatomical correlation and attenuation correction using BroadQuant (Siemens Healthcare GmBH, Erlangen, Germany).

### [^18^F]FDG PET/CT (response) scan

[^18^F]FDG PET/CT imaging was performed prior to and 9-weeks following initiation of anti-PD-1 with or without chemotherapy, according to local protocols and in accordance with current guidelines [18]. Patients were fasted at least 6 h and glucose levels confirmed <180 mg/dL prior to imaging. Patients were injected with a median activity of 331 MBq [^18^F]FDG (range 287–379) with images acquired at 60 min post-injection. Imaging was performed on a GE Discovery 710 PET/CT scanner with a 20-minute scan duration. A localisation and attenuation correction CT scan (140 kV, 10 mA, 0.5 s rotation time, and 40 mm collimation) started the imaging process. PET images were reconstructed using an iterative time-of-flight algorithm (2 iterations and 24 subsets) with a slice thickness of 3.27 mm and pixel size of 4.7 mm.

### CT (standard-of-care, TNM staging and response) scan

CT imaging was performed as standard-of-care diagnostic imaging prior to, and for response assessment at 9- and 18-weeks following, initiation of anti-PD-1 therapy with or without chemotherapy. Imaging at our institution was performed on a number of scanners (Siemens Definition Force, Siemens Definition Edge), all within local protocols (90–120 kVp with automated tube voltage selection, dose-modulated mA, 0.6 mm collimation, pitch 1.2, FOV 500 mm, matrix 512 × 512); CT scans were contrast-enhanced (Omnipaque 350 at a patient weight adapted dose of 2 mL/kg; at a rate of 3 mL/s with a 70-second delay), unless contraindicated, and reconstructed with soft tissue and lung kernels, with a minimum reconstructed slice thickness of 2 mm.

### Image analysis

CT images were analysed using PACS (SECTRA Medical) by an experienced radiologist with >20 years’ experience. TNM staging was performed on diagnostic/baseline CT imaging according to the 8th edition Lung Cancer Stage Classification [19]. Response at 9- and 18-weeks was assessed using RECIST v1.1 [20]. Up to 5 target lesions were identified at baseline and followed on sequential imaging. Partial response was defined as ≥ 30% decrease in the sum of diameters, whereas progressive disease was defined as ≥20% increase in the sum of diameters and/or presence of new lesion(s). Primary and metastatic lesions on CT were noted for subsequent identification on both SPECT/CT and PET/CT.

SPECT/CT and PET/CT images were analysed by an experienced nuclear medicine physician with >30 years’ experience using Hermes GOLD^TM^ software (Hermes Medical Solutions; Stockholm, Sweden). In attenuation corrected [^99m^Tc]NM-01 SPECT/CT images regions of interest (ROI) were placed on primary and metastatic lesions as well as mediastinal blood pool within the aortic arch to provide maximum uptake values (ROI_max_) for calculation of a tumour-to-blood pool ratio (T:BP) for each lesion. Regions of interest were identified from standard-of-care CT imaging but guided by the CT component of the SPECT/CT. Further details regarding the technique for image analysis with inter- and intra-observer validation have previously been reported [21].

Regions of interest correlating with standard CT imaging were also identified on attenuation corrected [^18^F]FDG PET/CT images and maximum standardised uptake values (SUV_max_) recorded. Responding patients were defined as those with a partial (PMR) or complete metabolic response (CMR) at 9-weeks following initiation of anti-PD-1 therapy, according to EORTC criteria with up to 5 target lesions identified at baseline and followed up on interval imaging [22]. Response was defined as ≥25% reduction in SUV_max_ from baseline, with progressive metabolic disease (PMD) defined as either a ≥ 25% increase in SUV_max_ from baseline across target lesions and/or presence of new lesions. Response in individual lesions was also defined as a ≥ 25% reduction in SUV_max_ from baseline.

### Statistical analyses

Continuous data are presented using descriptive statistics, including median values with their 95% confidence intervals (CI). Categorical data are presented as absolute and relative frequencies. Normality of continuous variables were assessed using the Shapiro-Wilk test. Correlations between two variables were calculated using Pearson’s correlation coefficient (*r*). Comparisons between unpaired continuous groups were made using the Mann-Whitney *U*-test. *P* values are one-sided with significance as α = 0.05. Statistical analyses were performed, and individual graphs created, using GraphPad Prism v10.1 for macOS (GraphPad Software, San Diego, California, USA). Figures were generated using BioRender.com.

## Results

Fifteen patients with advanced NSCLC scheduled for anti-PD-1 pembrolizumab (200 mg Q3W) with or without chemotherapy were recruited (Supplementary Fig. 1). Patient characteristics are summarised in Table 1 (full in Supplementary Table 1). Patients were median 63 years of age (range 53–75 years), 60% (*n* = 9) male, and 93% (*n* = 14) of white ethnicity. Fifty-three percent (*n* = 8) had PD-L1 TPS immunohistochemistry ≥ 50%, 13% (*n* = 2) had PD-L1 TPS of 1–49%, and 33% (*n* = 5) had PD-L1 TPS ≤ 1%. The median time from tissue biopsy to consent was 26 days (range 15–57) and to baseline [^99m^Tc]NM-01 SPECT/CT was 33 days (range 20–63). All patients were diagnosed with de novo advanced disease and had not received any systemic or radio-therapy prior to enrolment or biopsy that may have affected treatment response or PD-L1 immunohistochemistry, respectively. Forty-seven percent (*n* = 7) received pembrolizumab alone, with the remaining 53% (*n* = 8) receiving pembrolizumab with platinum-based cytotoxic chemotherapy. Thirteen patients completed both baseline and 9-weeks imaging; 2 patients died due to disease or immunotherapy related complications prior to the 9-weeks imaging.

**Table 1:** Summary of patient characteristics.

Clinical Characteristic	*n* = 15
*Age (years)*	
Median	63
Range	53–75
*Sex, n (%)*	
Female	6 (40)
Male	9 (60)
*Ethnicity, n (%)*	
White (British, Irish, other)	14 (93)
Black (African, British, Caribbean)	1 (7)
Asian (Asian, British)	0 (0)
other	0 (0)
*Smoking status, n (%)*	
Never smoker	1 (6)
Ex-smoker	10 (67)
Smoker	4 (27)
*ECOG PS, n (%)*	
0	4 (27)
1	11 (73)
*Histopathology, n (%)*	
NSCLC-non-squamous	12 (80)
NSCLC-squamous	3 (20)
*Tumour (T) stage, n (%)*	
X	1 (7)
1a	0 (0)
1b	2 (13)
1c	1 (7)
2a	2 (13)
2b	0 (0)
3	1 (7)
4	8 (53)
*Node (N) stage, n (%)*	
X	1 (7)
0	1 (7)
1	0 (0)
2	7 (46)
3	6 (40)
*Metastatic (M) stage, n (%)*	
M0 or Mx	1 (7)
M1a	5 (33)
M1b	6 (40)
M1c	3 (20)
*PD-L1 TPS (SP263), n (%)*	
< 1%	5 (33)
1–49%	2 (13)
≥50%	8 (53)
Not available	0 (0)
*Systemic anti-cancer therapy, n (%)*	
Single agent pembrolizumab	7 (47)
Pembrolizumab with chemo.	8 (53)
- *carboplatin + pemetrexed*	*6 (40)*
- *carboplatin + paclitaxel*	*2 (13)*
***[***^*99m*^*Tc]NM-01 dose (MBq), baseline*	
Mean	547
Median	541
Range	329–694
*[*^*99m*^*Tc]NM-01 dose (MBq), 9 weeks*	*(n* = *13)*
Mean	601
Median	629
Range	368–721
*[*^*18*^*F]FDG dose (MBq), baseline*	
Mean	337
Median	331
Range	294–379
*[*^*18*^*F]FDG dose (MBq), 9 weeks*	*(n* = *13)*
Mean	328
Median	324
Range	287–374

### [^99m^Tc]NM-01 uptake is heterogenous

**Fig. 1:**
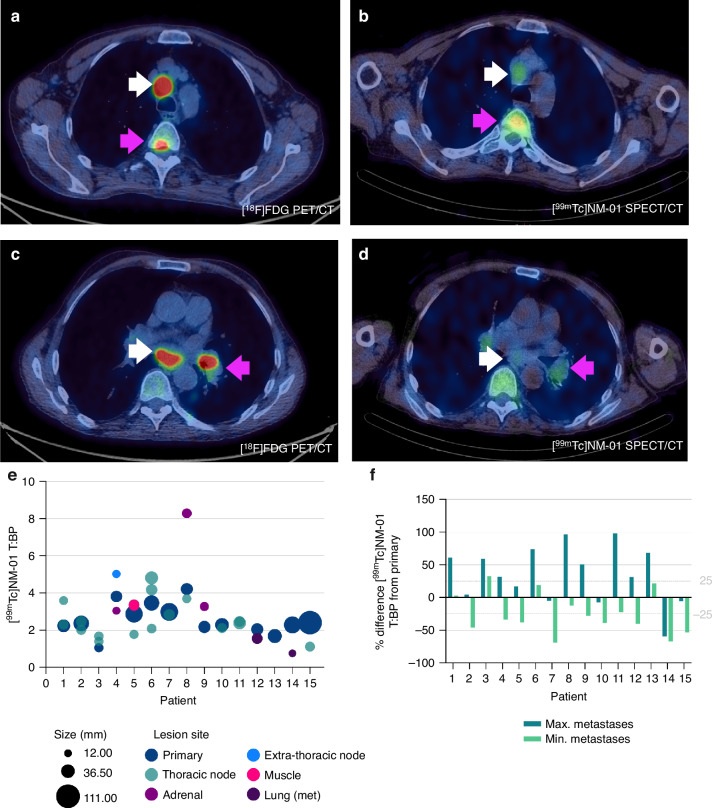
[^99m^Tc]NM-01 uptake in NSCLC demonstrates intertumoural heterogeneity. Baseline attenuation corrected and fused axial [^18^F]FDG PET/CT (left) and [^99m^Tc]NM-01 SPECT/CT (right) images (**a**–**d**) in a 63 year old male with PD-L1 positive metastatic NSCLC (PD-L1 TPS 100%, biopsy from station 2 R lymph node). **a** [^18^F]FDG PET/CT showing 2 R node with SUV_max_ 25.2 (white arrow) and T5 vertebra metastasis SUV_max_ 14.3 (pink arrow). **b** [^99m^Tc]NM-01 SPECT/CT with 2 R node T:BP of 3.88 (white arrow) and T5 vertebra metastasis T:BP 6.08 (pink arrow). **c** [^18^F]FDG PET/CT showing subcarinal node metastasis with SUV_max_ 29.9 (white arrow) and left hilar primary lesion SUV_max_ 29.6 (pink arrow). **d** [^99m^Tc]NM-01 SPECT/CT with subcarinal node metastasis with T:BP of 2.38 (white arrow) and left hilar primary lesion with T:BP of 3.07. **e** Interlesional heterogeneity of baseline [^99m^Tc]NM-01 T:BP within individual patients according to lesion location and size (mm), as determined with baseline CT imaging. **f** Bar chart showing the difference (%) between the [^99m^Tc]NM-01 T:BP of both the maximum (dark green) and minimum (light green) metastases from the primary tumour in all patients, with a ± 25% difference between the primary and at least one metastasis in 100% (*n* = 15) patients, and ± 50% difference in 67% (*n* = 10).

Interlesional heterogeneity was determined by measuring [^99m^Tc]NM-01 T:BP in both primary lung tumours (*n* = 15) and individual metastases (*n* = 56) in all patients. The median number of metastases delineated per patient was 4 (range 2–5). The median [^99m^Tc]NM-01 T:BP for primary tumour was 2.36 (interquartile range (IQR) 3.03–2.2), whilst median [^99m^Tc]NM-01 T:BP for metastases was 2.29 (IQR 3.29–1.66). Thoracic nodal metastases demonstrated a lower median [^99m^Tc]NM-01 T:BP at 2.28 (*n* = 38; IQR 2.94–1.79) compared to non-thoracic node metastases at 2.41 (*n* = 18; IQR 3.32–1.37). Within all individual patients, there was a greater than ±25% difference of [^99m^Tc]NM-01 T:BP measured between the primary tumour and either metastasis with the lowest (minimum) or highest (maximum) [^99m^Tc]NM-01 uptake (Fig. 1). In ten patients (67%), there was a greater than ±50% difference between the primary tumour and min/max metastasis. Heterogeneity between metastases only in individual patients was also demonstrated, with a minimum to maximum metastasis ratio of between 1.20 - 3.08.

### [^99m^Tc]NM-01 uptake correlates with PD-L1 immunohistochemistry

**Fig. 2:**
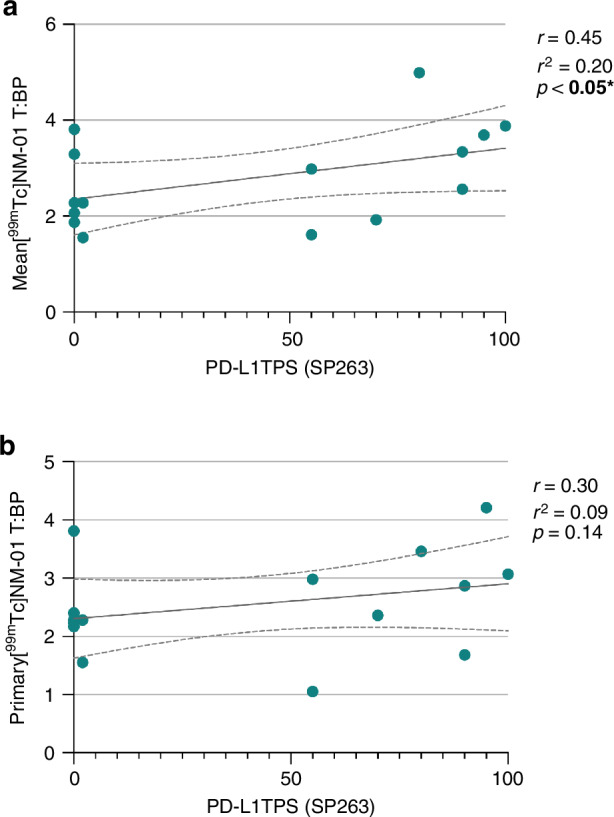
Baseline [^99m^Tc]NM-01 uptake correlates with PD-L1 immunohistochemistry. **a** Matched to histology site mean [^99m^Tc]NM-01 T:BP moderately correlates with PD-L1 TPS measured by immunohistochemistry (*n* = 15; *r* = 0.45, ***p*** < 0.05*). **b** Primary tumour [^99m^Tc]NM-01 T:BP (non-matched) demonstrated weak correlation with PD-L1 TPS (*n* = 15; *r* = 0.30, *p* = 0.14).

The mean [^99m^Tc]NM-01 T:BP was measured for corresponding lesion(s) sampled for measurement of PD-L1 TPS by immunohistochemistry. The matched mean [^99m^Tc]NM-01 T:BP demonstrated moderate correlation with PD-L1 TPS (correlation coefficient *r* = 0.45, *p* < 0.05; Fig. 2a). When using primary tumour [^99m^Tc]NM-01 T:BP, noting only 5/15 patients had matched immunohistochemistry of primary tumour, there was only weak correlation with PD-L1 TPS (*r* = 0.30, *p* = 0.14; Fig. 2b), in keeping with interlesional heterogeneity of expression.

### [^99m^Tc]NM-01 uptake is associated with early metabolic response

**Fig. 3:**
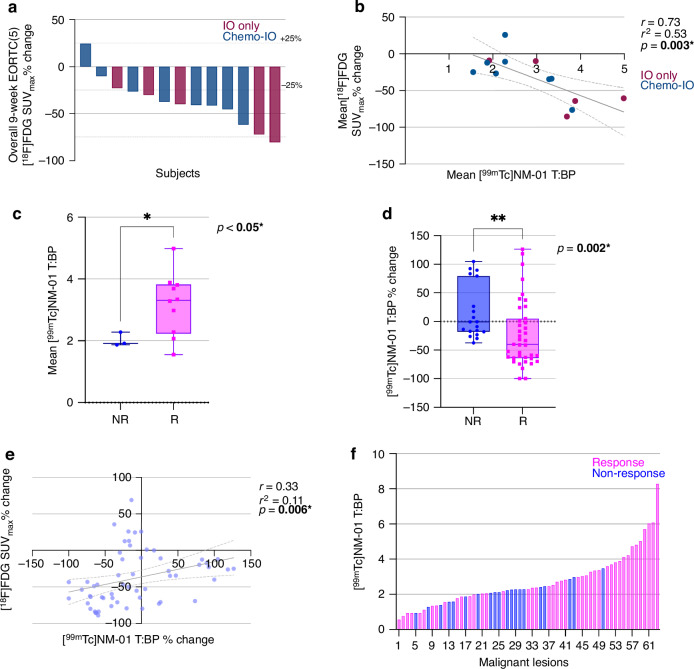
[^99m^Tc]NM-01 is associated with early metabolic response to anti-PD-1 therapy. **a** Waterfall plot of 9-week [^18^F]FDG SUV_max_ % change, with EORTC ± 25% indicated with dashed horizontal lines, and patients according to treatment, as anti-PD-1 alone (IO only; maroon) or combined with chemotherapy (chemo-IO; dark blue). **b** Higher baseline mean [^99m^Tc]NM-01 T:BP (i.e. PD-L1 expression) correlates with mean [^18^F]FDG SUV_max_ % change (response) (r = −0.73; p = 0.003*). **c** mean baseline [^99m^Tc]NM-01 T:BP according to 9-week EORTC defined response (*n* = 10; median 3.31; lower quartile 2.29, upper quartile 3.83; pink) vs non-response (*n* = 3; median 1.92; lower quartile 1.87, upper quartile 2.28; blue), Mann Whitney U-test *p* < 0.05*. **d** [^99m^Tc]NM-01 T:BP % change is significantly different in responders (*n* = 39; median −39.84; lower quartile −64.13, upper quartile 5.07; pink) vs non-responders (*n* = 19; median −0.67; lower quartile −17.92, upper quartile 79.37; blue), Mann Whitney U-test *p* = 0.002*. **e** Individual lesion [^18^F]FDG SUV_max_ % change from 0 to 9 weeks demonstrates weak correlation with [^99m^Tc]NM-01 T:BP % change (*r* = 0.33; *p* = 0.006*). **f** Individual lesions (*n* = 63) [^99m^Tc]NM-01 T:BP displayed according to response (pink) and non-response (blue), demonstrating higher [^99m^Tc]NM-01 T:BP is associated with response status. For all boxplots, the horizontal line within the boxplot indicates the median, with the lower edge representing the lower quartile, and upper edge the upper quartile. Whiskers represent the minimum and maximum values.

Metabolic response, determined by EORTC criteria of [^18^F]FDG PET/CT (mean ≥ 25% reduction in up to 5 lesions including primary) was present in 10/13 patients (Fig. 3a). Of these, 40% (*n* = 4) received pembrolizumab alone. Of the 3 non-responders, 1 patient received pembrolizumab alone. Baseline mean [^99m^Tc]NM-01 T:BP, representative of PD-L1 expression, was significantly higher in patients with an [^18^F]FDG PET/CT metabolic response, according to EORTC criteria, at 9-weeks. The mean [^99m^Tc]NM-01 T:BP in responding patients was 3.19 (*n* = 10; 95% CI 2.46–3.91) compared to that of non-responding patients at 2.02 (*n* = 3; 95% CI 1.47–2.58; ***p*** < 0.05*) (Fig. 3b). This was in contrast to PD-L1 TPS, which was not statistically different between [^18^F]FDG PET/CT responders (*n* = 10; mean 42.40%; 95% CI 9.90–74.90) and non-responders (*n* = 3; mean 23.33%; 95% CI −77.06 to123.70; *p* = 0.17) (Supplementary Fig. 2a). Importantly, baseline mean [^99m^Tc]NM-01 T:BP was also significantly higher in patients with partial response at 3.79 (*n* = 3; 95% CI 3.55–4.03) compared to non-responders (stable or progressive disease) at 2.56 (*n* = 11; 95% CI 1.88–3.25), determined by CT RECIST v1.1 criteria at 9-weeks (***p*** < 0.05*). However, the difference was not significantly different for response at 18-weeks determined by CT (Supplementary Fig. 3).

**Fig. 4:**
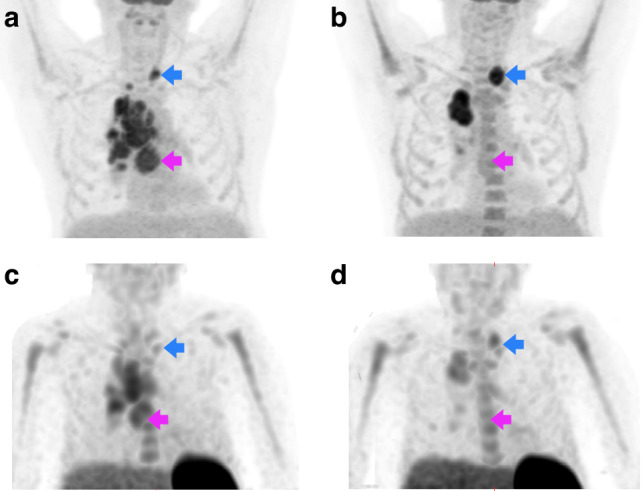
Changes in [^99m^Tc]NM-01 uptake may be associated with metabolic response. Maximum intensity projection images of (**a**) baseline and (**b**) 9-weeks [^18^F]FDG PET/CT and (**c**) baseline and (**d**) 9-weeks [^99m^Tc]NM-01 SPECT/CT in a 64 year old male with PD-L1 positive (PD-L1 TPS 80%; from stations 4 R, 4 L and 7 thoracic lymph nodes) metastatic NSCLC and dissociated metabolic response at 9-weeks following single agent anti-PD-1 pembrolizumab. For example, a subcarinal (station 7) node metastasis responds to treatment, with a baseline (**a**) [^18^F]FDG SUV_max_ of 9.1 and (**c**) [^99m^Tc]NM-01 T:BP of 4.81, and 9-weeks (**b**) SUV_max_ 3.4 and (**d**) [^99m^Tc]NM-01 T:BP of 1.41 (pink arrows). However, a left supraclavicular fossa node does not respond to treatment, with a baseline (**a**) [^18^F]FDG SUV_max_ of 8.5 and (**c**) [^99m^Tc]NM-01 T:BP of 2.08, and 9-weeks (**b**) SUV_max_ 12.2 and (**d**) [^99m^Tc]NM-01 T:BP of 2.74 (blue arrows).

Additionally, mean baseline [^99m^Tc]NM-01 T:BP strongly correlated with mean [^18^F]FDG PET/CT SUV_max_ %change (reduction) at 9-weeks (*n* = 13; *r* = −0.73; *p* = 0.003) (Fig. 3c). Whilst PD-L1 TPS only moderately correlated with mean [^18^F]FDG PET/CT SUV_max_ %change (*n* = 13; *r* = −0.46; *p* = 0.06) (Supplementary Fig. 2b). There was also a weak but significant correlation of [^18^F]FDG PET/CT SUV_max_ %change with [^99m^Tc]NM-01 T:BP %change from 0 to 9-weeks for all lesions (n = 58; *r* = 0.33; *p* = 0.006) (Fig. 3d). The mean [^99m^Tc]NM-01 T:BP %change was greater in responding lesions at −21.90 (*n* = 39; 95% CI −40.63 to −3.16) compared to non-responding lesions at 16.29 (*n* = 19; 95% CI −6.84 to 39.42; *p* = 0.002) (Fig. 3e). An example case demonstrating both responding and non-responding lesions is presented (Fig. 4).

## Discussion

In this study, we demonstrated that non-invasive quantification of PD-L1 with [^99m^Tc]NM-01 SPECT/CT may determine spatial and temporal heterogeneity of PD-L1 expression and potentially may predict early metabolic response to treatment with anti-PD-1 therapy with/without chemotherapy in advanced NSCLC. [^99m^Tc]NM-01 uptake was measurable in all patients, with ≥25% heterogeneity between primary tumour and at least one metastasis in all cases. This further supports the need for a novel method, beyond single-site histology, that allows whole-body PD-L1 quantification for optimal biomarker predictive value and therapeutic utility.

Although the correlation of mean matched [^99m^Tc]NM-01 T:BP with PD-L1 TPS determined by immunohistochemistry was statistically significant, it was moderately so, with a correlation coefficient (*r*) of 0.45. As PD-L1 heterogeneity is well-documented, excellent correlation would not be expected unless non-invasive quantification with [^99m^Tc]NM-01 T:BP was no better than standard immunohistochemistry. PD-L1 TPS is the recognised and approved scoring approach in NSCLC but only takes into account tumour cell expression, whilst PD-L1 imaging techniques, including [^99m^Tc]NM-01 SPECT/CT, quantify PD-L1 expression in the entire tumour microenvironment. The combined positive score (CPS), which takes into account expression on both tumour and immune cells, may therefore be a more suitable comparator, however, it is not used clinically nor routinely in NSCLC, limiting its applicability and the interpretation of associated response data.

An alternative approach to PD-L1 imaging has been to radiolabel anti-PD-(L)1 drugs, such examples being [^89^Zr]durvalumab and [^89^Zr]atezolizumab, with uptake demonstrated in a range of malignancies [23, 24]. A study of 22 patients with advanced NSCLC, breast, or bladder cancer, demonstrated heterogenous [^89^Zr]atezolizumab uptake, SUV_max_ measured with PET/CT, within and between patients, and uptake above the mean was associated with improved progression free and overall survival with anti-PD-L1 therapy [24]. Whilst there was a trend to increasing [^89^Zr]atezolizumab uptake with PD-L1 SP142 immunohistochemistry score (*p* = 0.048), there was no difference in the SUV_max_ according to PD-L1 SP263 positive vs negative status (*p* = 0.15). It is important to note, however, that the level for PD-L1 positivity in this study using the SP263 assay was set at ≥25%, which is not a clinically validated cut-off for anti-PD-(L)1 therapies in NSCLC. A potential benefit of ‘drug’ radiopharmaceuticals is that it can also determine in vivo drug distribution and has theranostic potential due to the high affinity and specificity of monoclonal antibodies. However, a significant limitation is the large size of monoclonal antibodies (often around 150 kDa), with adequate biodistribution for optimal tumour-to-background contrast in the region of many hours to days [25]. This restricts the potential radiotracers for labeling, which are currently expensive and have reduced availability in many countries, and along with the delay between injection and imaging, requiring patient isolation and environmental radiation safety concerns and measures, limit their potential for routine clinical use. It also remains unclear how to interpret changes in uptake of drug radiopharmaceuticals on longitudinal imaging when therapeutic drug is on board, with direct competition for PD-L1 binding. Radiolabeled small antibody fragments or peptides may provide a potential solution, such as the [^18^F] labeled, synthetically engineered, adnectin monobody ( ~ 10 kDa) to PD-L1 called BMS-986192 [26]. In a study of 9 patients with advanced NSCLC, [^18^F]BMS-986192 uptake, measured with PET/CT at 1 hr post-injection, was heterogeneous with higher median SUV_peak_ in patients with high PD-L1 TPS ≥ 50% (*p* = 0.018) [26]. Similarly, [^99m^Tc]NM-01, a radiolabeled sdAb ( ~ 15 kDa) radiopharmaceutical, displays rapid biodistribution, with optimal SPECT/CT imaging at 2 h post-injection for same-day imaging, thus enhancing the potential clinical application and utility of non-invasive PD-L1 imaging [16]. Importantly it also has a different binding domain to therapeutic anti-PD-L1 antibodies, ensuring that [^99m^Tc]NM-01 can bind to and allow quantification of PD-L1 longitudinally despite anti-PD-L1 therapy [13].

Interestingly, in this study, we found that PD-L1 expression, determined by [^99m^Tc]NM-01 T:BP, can display temporal heterogeneity, and that a larger difference in [^99m^Tc]NM-01 T:BP between 0 and 9-weeks was associated with a deeper metabolic response. The mechanism for this is unclear, but could represent a true reduction of cells within the tumour microenvironment of the responding lesion(s) for [^99m^Tc]NM-01 to bind to. Alternatively, this could represent a reduction in PD-L1 expression by cancer cells due to altered immune checkpoint signaling, secondary to anti-PD-(L)1 therapy. PD-L1 expression is also closely linked to glycolysis, HIF-1a and GLUT1 expression, hence a responding tumour with less glycolytic activity may potentially result in PD-L1 downregulation [27–29]. This requires further evaluation in both pre-clinical and clinical studies.

Improving the predictive value of PD-L1 expression for anti-PD-(L)1 therapies at baseline is an obvious and important potential use for non-invasive PD-L1 assessment. However, given that PD-L1 expression also demonstrates temporal heterogeneity, longitudinal non-invasive imaging, with for example [^99m^Tc]NM-01 SPECT/CT, may play an important role clinically. One such example would be in the case of dissociated response, where some malignant lesions progress despite response in a majority of other lesions. Repeat PD-L1 imaging could for example demonstrate increasing PD-L1 expression in the progressing lesions despite anti-PD-(L)1 therapy, allowing the oncologist to consider the addition of cytotoxic chemotherapy or localised radiotherapeutic approaches, whilst maintaining immune anti-PD-(L)1 therapy. This is certainly an attractive option, given that cytotoxic chemotherapy and radiotherapy can work synergistically with immune checkpoint inhibitors, stimulating an immune response through the release of neoantigens [30, 31]. Such novel approaches, directing therapeutic decisions based on PD-L1 imaging and response certainly warrant further investigation in clinical trials. For example, further insight into the potential utility of non-invasive PD-L1 assessment, within the context of radiotherapy, may come from an ongoing clinical trial of [^89^Zr]durvalumab in stage III NSCLC patients undergoing chemoradiation (ACTRN12621000171819) [32].

There are some limitations to acknowledge in this study. Firstly, this is a single-centre study with a relatively small sample size. The study only includes patients with advanced NSCLC, and as such, its reproducibility in other cancers needs validation. PD-L1 CPS could not reliably be performed retrospectively in our cohort, which included lymph node samples. Given that quantification of PD-L1 radiopharmaceuticals would include both tumour and immune cell labeling, the CPS is likely a better comparator and we plan to investigate this prospectively in future clinical studies. In this study, we investigated the association of [^99m^Tc]NM-01 uptake with metabolic response determined with [^18^F]FDG PET/CT at 9-weeks. Here, we demonstrated the association with metabolic response, however, it is unclear from this early timepoint if this would translate to objective response, and longer term survival in this heterogenous NSCLC cohort receiving immunotherapy with/without chemotherapy. This requires further investigation with standard CT RECIST and [^18^F]FDG PET/CT response evaluation criteria at recognised 12 weekly intervals within a phase III study. Brain imaging was not performed in this study, so it is unknown whether there is adequate blood-brain-barrier penetrance of [^99m^Tc]NM-01 and demonstratable uptake in brain metastases. Also, whilst SPECT/CT provides benefits in terms of availability, access and cost, there are physical limitations with regards to spatial resolution and susceptibility to partial volume effect. This may be overcome with further novel methods and validation of quantifiable SPECT/CT. Despite this limitation, our imaging demonstrated good tumour-to-background contrast, with readily quantifiable [^99m^Tc]NM-01 uptake. This requires validation in further studies, but suggests NM-01 has the potential to be a PD-L1 imaging agent, with widespread clinical applications, as well as in pre-clinical research. Further validation of the association and heterogeneity of [^99m^Tc]NM-01 uptake with PD-L1 immunohistochemistry could be made using contemporaneous SPECT/CT and biopsy and/or resection samples, as well as with autoradiography.

## Conclusions

This study demonstrates that PD-L1 imaging, using the [^99m^Tc]-labeled anti-PD-L1 sdAb, NM-01, with SPECT/CT is feasible in advanced NSCLC. [^99m^Tc]NM-01 uptake moderately correlated with PD-L1 immunohistochemistry, demonstrated intertumoural heterogeneity, and was associated with early metabolic response to anti-PD-1 therapy. PD-L1 expression determined by [^99m^Tc]NM-01 SPECT/CT imaging has the potential to better predict response compared to PD-L1 TPS measured with immunohistochemistry. [^99m^Tc]NM-01 SPECT/CT may therefore play an important future role in both the diagnostic and treatment pathway of NSCLC. Non-invasive and longitudinal assessment of PD-L1 has the potential to improve PD-L1 predictive value, and direct novel approaches to both clinical trial design and therapy. Further validation is now warranted with an expanded phase III clinical trial.

## Supplementary information

Supplemental material

## Data Availability

The data used and/or analyzed in this study are available from the corresponding author on reasonable request.
