# Supplementary material for: PD-L1 imaging with [99mTc]NM-01 SPECT/CT is associated with metabolic response to pembrolizumab with/without chemotherapy in advanced lung cancer
Source: Br J Cancer. 2025 Apr 5;132(10):913–21. doi: 10.1038/s41416-025-02991-w (PMC12081918; doi:10.1038/s41416-025-02991-w)
Supplement: Supplementary file 1 — Supplemental material [file 41416_2025_2991_MOESM1_ESM.docx]

**SUPPLEMENTARY MATERIAL**

**
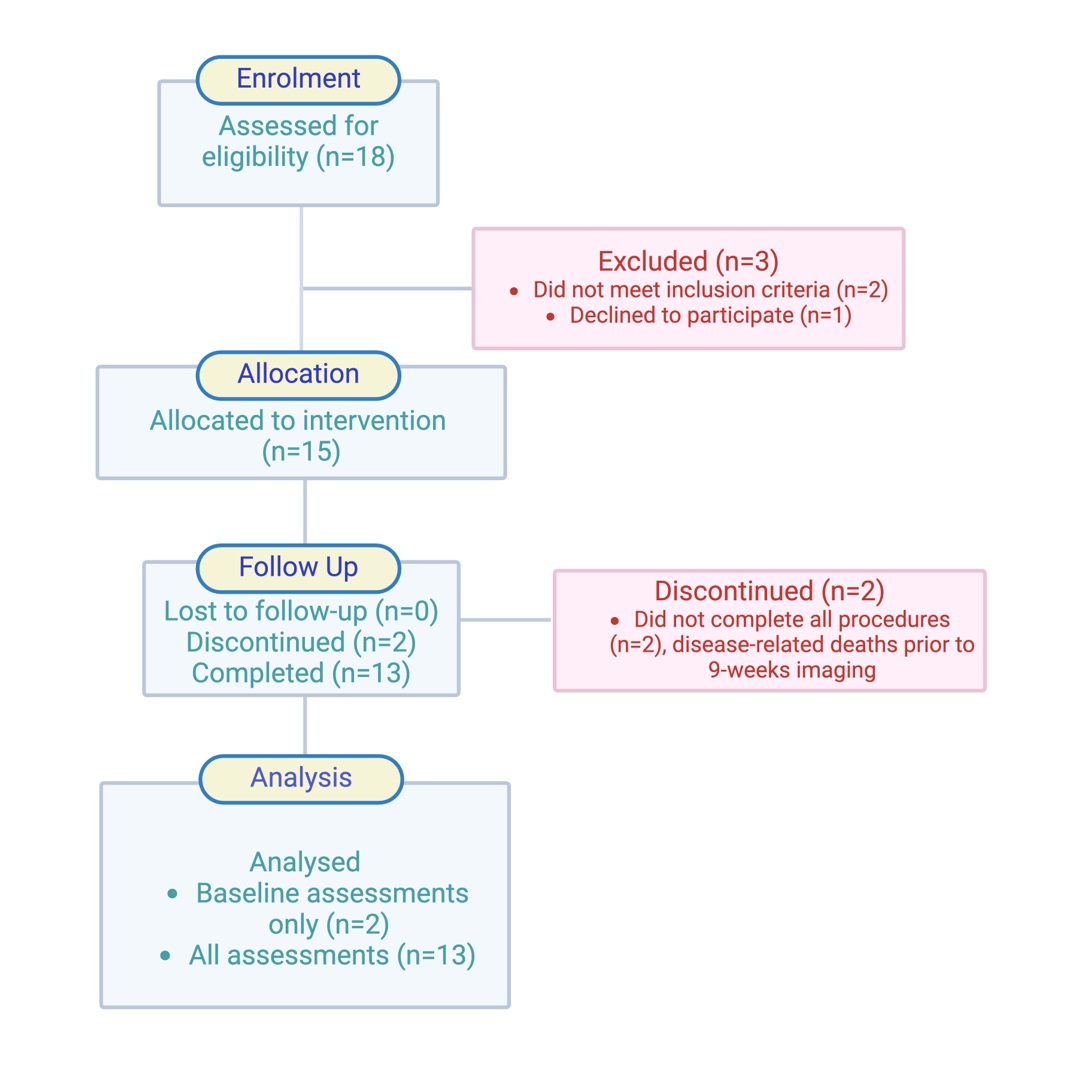
**

***Supplementary Figure 1. CONSORT diagram detailing study recruitment.***


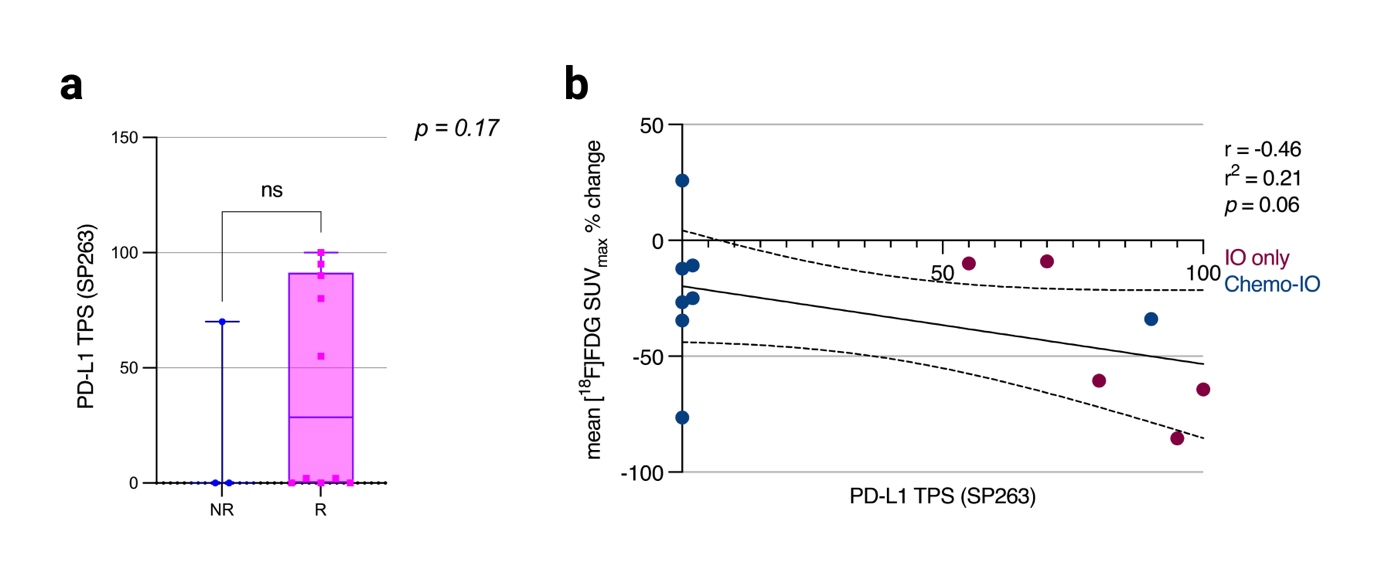


***Supplementary Figure 2. Baseline PD-L1 TPS measured by immunohistochemistry moderately correlates with early metabolic response, but does not differentiate between responders and non-responders.*** *(****a****) PD-L1 TPS immunohistochemistry was not significantly different between [^18^F]FDG PET/CT responders (n=10; median 28.50; lower quartile 0.00, upper quartile 91.25) and non-responders (n=3; median 0.00; lower quartile 0.00, upper quartile 70.00; p = 0.17). (****b****) PD-L1 TPS moderately correlates with mean [^18^F]FDG PET/CT SUV_max_ %change (n=13; r = -0.46; p = 0.06). Horizontal line within the boxplots indicate the median, with the lower edge representing the lower quartile, and upper edge the upper quartile. Whiskers represent the minimum and maximum values.*


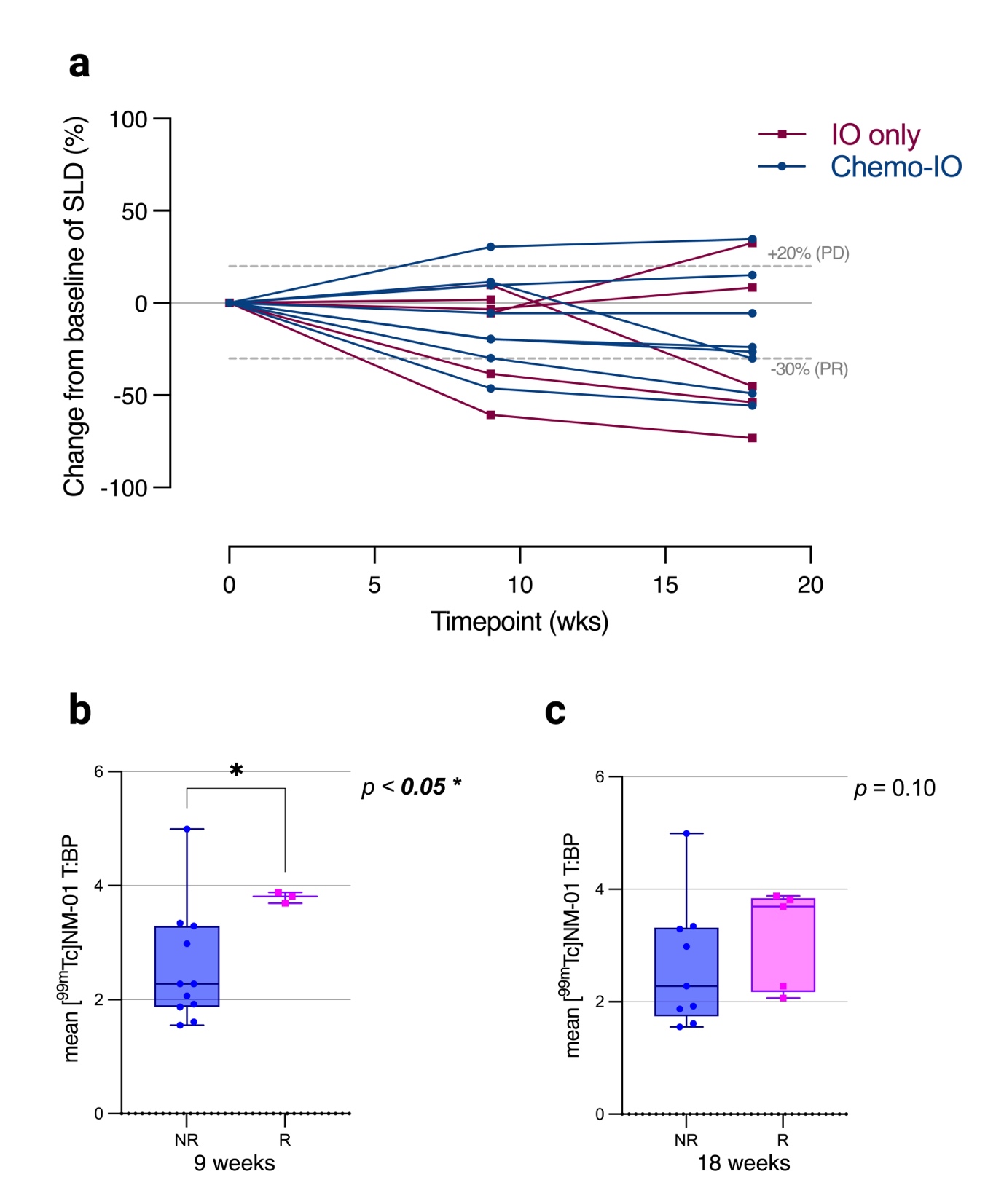


***Supplementary Figure 3. Baseline mean [^99m^Tc]NM-01***  ***T:BP measured by SPECT/CT is associated with early 9-weeks response by CT RECIST criteria, but not at 18-weeks.*** *(****a****) Spaghetti plot demonstrating the change from baseline (%) in the sum of longest diameter (SLD) at 9 and 18 weeks following anti-PD-1 with/without chemotherapy as per CT RECIST v1.1 criteria; +20% indicates progression of disease, whereas -30% indicates partial response (note 1 patient met criteria for PD on basis of new metastasis rather than SLD ≥20%; 1 patient did not have follow-up CTs at 9 or 18 weeks due to immunotherapy toxicity-related death). (****b****) The baseline mean [^99m^Tc]NM-01 T:BP was significantly higher in those patients (n=3) with response (PR) at 3.79 (95% CI 3.55 - 4.03) vs those patients (n=11) with no response (either SD or PD) at 2.56 (95% CI 1.88 - 3.25), at 9 weeks determined by CT RECIST v1.1 (****p = 0.02****).*

*(****c****) However, the baseline mean [^99m^Tc]NM-01 T:BP was no different in those patients (n=5) with response (PR) at 3.15 (95% CI 2.04 - 4.25) vs those patients (n=9) with no response (either SD or PD) at 2.65 (95% CI 1.79 - 3.51), at 18 weeks determined by CT RECIST v1.1 (p = 0.10). Horizontal line within the boxplots indicate the median, with the lower edge representing the lower quartile, and upper edge the upper quartile. Whiskers represent the minimum and maximum values. IO-only denotes immunotherapy only, whilst Chemo-IO denotes combination chemotherapy and immunotherapy. PR denotes partial response, SD is stable disease, and PD is progressive disease.*

| Patient no. | Age (years) | Sex | Ethnicity | Smoker status | ECOG PS | Histopath-ology | SACT | TNM stage | SP263 PD-L1 TPS, % (group) | 22C3 PD-L1 TPS, % (group) | *[^99m^Tc]NM-01 dose (MBq), baseline* | *[^99m^Tc]NM-01 dose (MBq),*  *9 weeks* | *[^18^F]FDG dose (MBq), baseline* | *[^18^F]FDG dose (MBq),*  *9 weeks* |
| --- | --- | --- | --- | --- | --- | --- | --- | --- | --- | --- | --- | --- | --- | --- |
| 1 | 63 | M | White - British | Ex | 0 | NSCLC-adeno | pembrolizumab + carbo/pem | TX N2 M1a | 0  (<1) | 0  (<1) | 689 | 618 | 361 | 297 |
| 2 | 59 | M | White - other | Ex | 1 | NSCLC-adeno | pembrolizumab | T4 N3 M0 | 70 (≥50) | 40  (1-49) | 512 | 713 | 350 | 310 |
| 3 | 53 | F | White - British | Ex | 0 | NSCLC-adeno | pembrolizumab | T4 N3 M1c | 55  (≥50) | -- | 547 | -- | 326 | -- |
| 4 | 66 | F | White - British | Ex | 0 | NSCLC-adeno | pembrolizumab + carbo/pem | T1b N0 M1b | 0  (<1) | 0  (<1) | 694 | 706 | 324 | 324 |
| 5 | 58 | F | White - British | Ex | 1 | NSCLC-adeno | pembrolizumab + carbo/pem | T3 NX M1b | 90  (≥50) | 65  (≥50) | 541 | 574 | 332 | 287 |
| 6 | 64 | M | White - Irish | Ex | 1 | NSCLC-NOS | pembrolizumab | T4 N3  M1a | 80  (≥50) | 75  (≥50) | 644 | 721 | 379 | 350 |
| 7 | 73 | M | White - British | Ex | 1 | NSCLC-squam | pembrolizumab | T4 N2 M1b | 55  (≥50) | 5  (1-49) | 677 | 711 | 334 | 353 |
| 8 | 72 | M | White - British | Ex | 1 | NSCLC-adeno | pembrolizumab | T1b N2 M1b | 95  (≥50) | 95  (≥50) | 618 | 694 | 368 | 312 |
| 9 | 75 | F | White - other | Ex | 1 | NSCLC-adeno | pembrolizumab + carbo/pem | T2a N2 M1b | 0  (<1) | 0  (<1) | 538 | 689 | 331 | 336 |
| 10 | 59 | M | White - other | Smoker | 0 | NSCLC-adeno | pembrolizumab + carbo/pem | T2a N2 M1a | 0  (<1) | 0  (<1) | 658 | 425 | 362 | 295 |
| 11 | 63 | M | White - British | Smoker | 1 | NSCLC-NOS | pembrolizumab | T1c N3 M1c | 100  (≥50) | 50  (≥50) | 343 | 429 | 294 | 374 |
| 12 | 71 | M | White - British | Smoker | 1 | NSCLC-squam | pembrolizumab  + carbo/pacli | T4 N2 M1a | 2  (1-49) | 0  (<1) | 521 | 629 | 308 | 350 |
| 13 | 66 | F | White - British | Smoker | 1 | NSCLC-adeno | pembrolizumab | T4 N3 M1c | 90  (≥50) | 90  (≥50) | 362 | -- | 330 | -- |
| 14 | 54 | M | White - British | Ex | 1 | NSCLC-adeno | pembrolizumab  + carbo/pem | T4 N2 M1a | 2  (1-49) | 3  (1-49) | 528 | 531 | 322 | 320 |
| 15 | 53 | F | Black – other | Never | 1 | NSCLC-squam | pembrolizumab + carbo/pacli | T4 N3 M1b | 0  (<1) | 0  (<1) | 329 | 368 | 329 | 360 |

***Supplementary Table 1. Patient characteristics.*** *Adeno - adenocarcinoma; carbo - carboplatin; ECOG - Eastern Cooperative Oncology Group; F = female; M - Male; NSCLC (NOS) - non-small cell lung cancer (not otherwise specified); pacli - paclitaxel; PD-L1 - programmed death-ligand 1; pem - pemetrexed; SACT - systemic anti-cancer therapy; squam - squamous cell carcinoma; TNM - tumor nodal metastasis (stage); TPS - tumor proportion score.*
